# Supplementary material for: Health-Related Quality of Life following Cytoreductive Radical Prostatectomy in Patients with De-Novo Oligometastatic Prostate Cancer
Source: Cancers (Basel). 2021 Nov 11;13(22):5636. doi: 10.3390/cancers13225636 (PMC8616367; doi:10.3390/cancers13225636)
Supplement: Supplementary file 1 [file cancers-13-05636-s001.zip › cancers-1460763-supplementary.pdf]

Article

Health-related quality of life following cytoreductive radical prostatectomy in patients with de-novo oligometastatic prostate cancer

Michael Chaloupka[1], Lina Störmer[1], Maria Apfelbeck[1], Alexander Buchner[1], Christian G. Stief[1], Thilo Westhofen[1,*], Alexander Kretschmer[1,*]

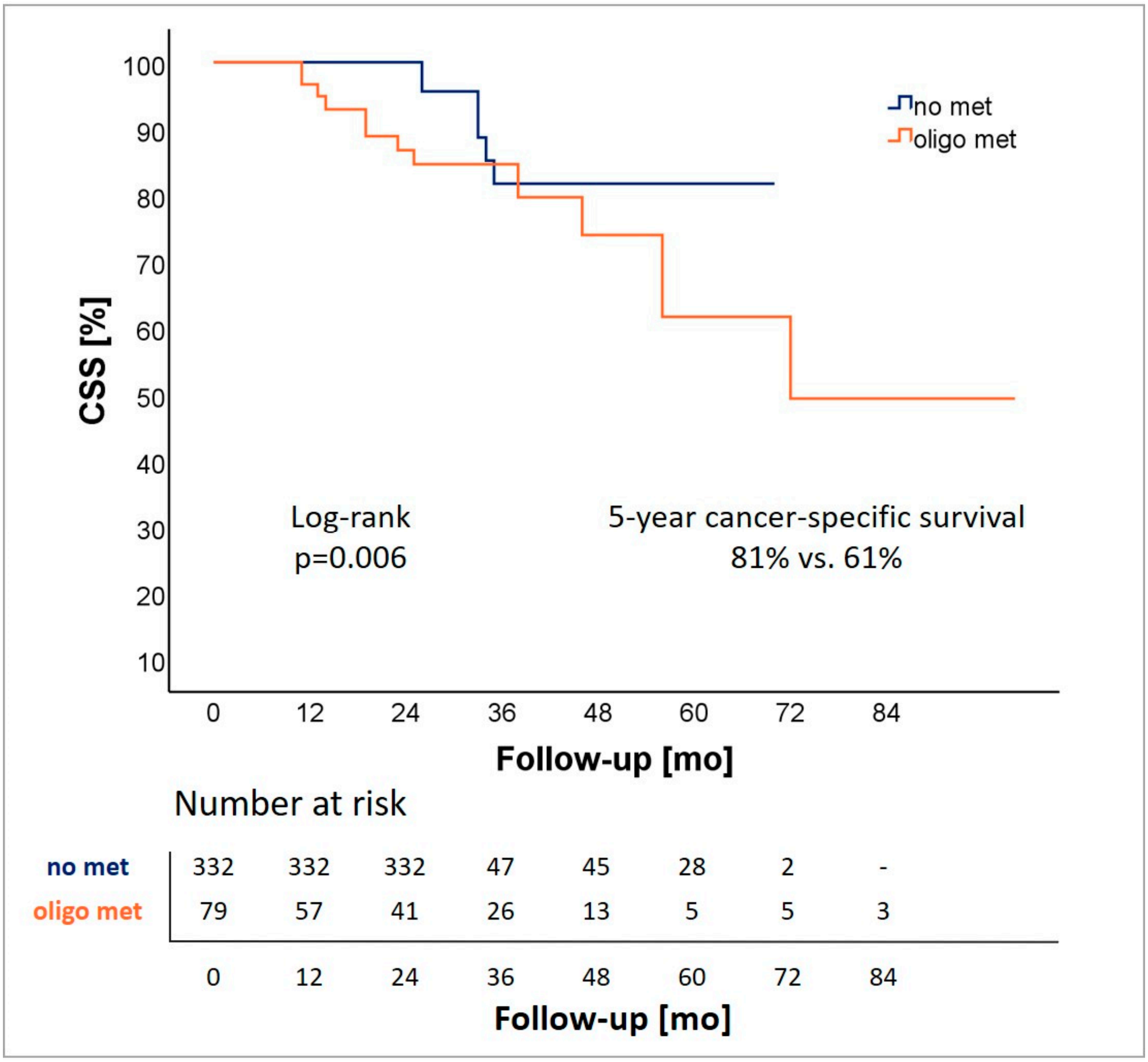

Figure S1a. Estimated 5-year cancer-specific survival (CSS).

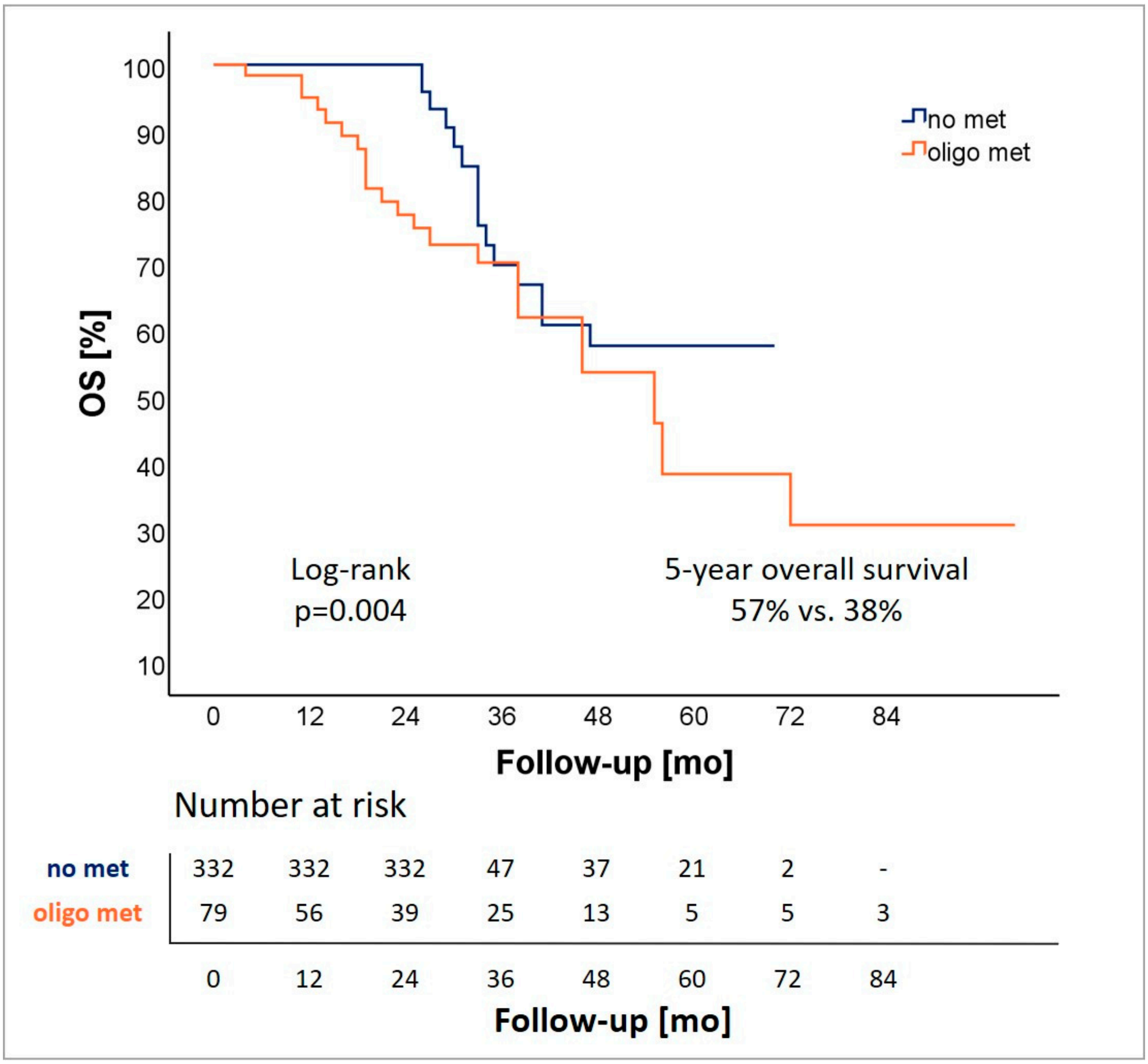

Figure S1b. Estimated 5-year overall survival (OS).

Table S1. Numerical analysis of net baseline changes of HRQOL pre- and postoperatively. Bold values indicate p values <0.05 and were considered statistically significant. Net change to preoperative status is presented HRQOL: Health-related quality of life; QLQ-C30: European Organization for Research and Treatment of Cancer quality of life questionnaire-Cancer 30.

		QLQ Net Baseline Change				
			**cM1-oligo**	***p***	**cM0**	***p***
	**Symptom scale**					
		Dyspnoea	5.1	0.165	12.4	***<0.001***
		Pain	-2.1	0.847	4.1	***0.044***
		Fatigue	8.3	***0.041***	11.8	***<0.001***
		Insomnia	5.6	0.439	9.1	***<0.001***
		Appetite loss	2.7	0.533	1.1	0148
		Nausea/vomiting	1.3	0.062	2.5	***0.001***
**QLQ - C30**		Constipation	7.7	0.036	5.5	***0.001***
		Diarrhoea	5.3	0.191	5.1	***0.002***
	**Financial difficulty scale**		2.1	0.746	5.3	***<0.001***
	**Functioning scale**					
		Physical	-11	***0.015***	-7.6	***<0.001***
		Role	-8.1	0.099	-14.9	***<0.001***

Cognitive	-5.9	0.309	-4.3	***0.005***
Emotional	1.6	0.796	0.8	0.470
Social	-5.5	0.392	-12.3	***<0.001***
**Global health status**	3.2	0.381	-5	***0.001***

Table S2. Longitudinal analysis of postoperative patient-reported HRQOL, assessed by the validated EORTC QLQ-C30 questionnaire. Bold values indicate *p* values <0.05 and were considered statistically significant. Continuous values are presented as median and standard-deviation (SD). HRQOL: Health-related quality of life; EORTC-QLQ-C30: European Organization for Research and Treatment of Cancer quality of life questionnaire-Cancer 30; QLQ-PR25: European Organization for Research and Treatment of Cancer quality of life questionnaire-Prostate 25.

Mean (SD) EORTC QLQ C30 score																
		T0			3M			12M			24M			36M		
		cM1-oligo	cM0	p	cM1-oligo	cM0	p	cM1-oligo	cM0	p	cM1-oligo	cM0	p	cM1-oligo	cM0	p
QLQ - C30	Symptom scale															
	Dyspnoea	7.1 (16.3)	7.7 (18.7)	0,959	11.1 (20.6)	6.3 (27.8)	0,530	6.7 (13.7)	19 (28)	0,067	14 (23.1)	18.7 (27.1)	0,757	15.4 (22)	19 (26.9)	0,783
	Pain	18.4 (27.0)	11.2 (20.7)	0,031	13.3 (20.1)	15.1 (19.9)	0,670	16.7 (16.2)	12.8 (21.7)	0,072	16.7 (25.5)	13.7 (23)	0,455	10.3 (19.9)	14.5 (24.6)	0,658
	Fatigue	23.6 (26.4)	13.7 (16.7)	0,012	29.6 (26.8)	30.4 (21.8)	0,753	30.3 (22.5)	26.7 (23.7)	0,382	36.8 (28.5)	24.5 (24.9)	0,024	34.2 (21)	25.6 (26.3)	0,109
	Insomnia	23.0 (33.9)	18.7 (26.1)	0,706	31.1 (38.8)	27.7 (31.2)	0,910	28.3 (31.1)	29.3 (31.9)	0,966	24.6 (31.1)	28.3 (30.6)	0,963	30.8 (39.6)	31.4 (32.8)	0,759
	Appetite loss	4.8 (13.4)	3.3 (12.7)	0,250	11.1 (20.6)	6.5 (15.7)	0,314	5 (12.2)	3.7 (11.7)	0,484	8.8 (18.7)	4.7 (13.8)	0,175	5.1 (18.5)	7.4 (20)	0,530
	Nausea/vomiting	0.1 (2.2)	1.0 (4.2)	0,069	3.3 (9.3)	3.9 (8.6)	0,638	1.7 (5.1)	2 (6.6)	0,993	0.9 (3.8)	3.1 (9.8)	0,345	1.3 (4.6)	2.5 (7.9)	0,696
	Constipation	7.9 (23.1)	5.7 (16.4)	0,949	6.7 (13.8)	13.3 (22.1)	0,347	18.3 (25.3)	9.6 (18)	0,098	24.6 (31.1)	10.2 (21.1)	0,004	10.3 (28.5)	12.4 (23.7)	0,456
	Diarrhoea	7.6 (18.9)	6.9 (15.7)	0,752	15.6 (27.8)	10.3 (20.8)	0,424	13.3 (19.9)	12.4 (21.6)	0,663	10.5 (15.9)	12.5 (21.7)	0,539	0 (0)	11.9 (21)	0,026
	Financial difficulty scale	11.1 (23.8)	2.7 (10.7)	<0.001	24.4 (29.5)	16.7 (27.1)	0,197	11.7 (19.6)	11.2 (22.9)	0,827	14 (25.6)	9 (20.3)	0,370	20.5 (34.8)	9.8 (21.3)	0,281
	Functioning scale															
	Physical	90.4 (15.6)	93.7 (12.7)	0,130	84.1 (17.6)	87.7 (14.9)	0,552	82.7 (22.7)	86.9 (17.2)	0,304	78.9 (22.4)	86.8 (16.8)	0,223	80 (26.5)	86 (18.5)	0,655
	Role	83.6 (26.4)	91.9 (19.4)	0,004	73.3 (28.7)	69.1 (26.0)	0,445	80.8 (23.1)	74.7 (25.7)	0,496	64.9 (38.4)	78.1 (26.5)	0,165	78.2 (32.9)	80.3 (27)	0,908
	Cognitive	86.8 (18.3)	89.6 (16.6)	0,272	74.4 (33.3)	88.1 (19.2)	0,192	86.7 (22)	87.1 (16)	0,405	81.6 (26.6)	85.9 (18.7)	0,944	82.1 (22)	83.6 (20.3)	0,853
	Emotional	65.8 (24.3)	74.2 (20.9)	0,014	61.7 (28.1)	72.0 (22.1)	0,185	72.1 (18.4)	75 (20.6)	0,422	64.9 (22.1)	75.8 (22)	0,040	67.9 (25.2)	77.7 (22.3)	0,150
	Social	77.2 (25.1)	85.5 (21.6)	0,007	73.3 (28.7)	64.9 (25.3)	0,148	68.3 (23.5)	71.8 (26.4)	0,632	64.9 (33.7)	74.3 (27.9)	0,143	73.1 (32.3)	75.7 (27.6)	0,912
	Global health status	63.6 (20.1)	71.8 (20.7)	0,004	65 (18.9)	64.7 (20.6)	0,983	64.2 (20.9)	71.5 (19.2)	0,027	65.7 (22.7)	67.1 (21.5)	0,704	64.7 (25.6)	70.3 (20.6)	0,334
QLQ - PR25	Urinary symptoms				32 (17.3)	36.2 (19.9)	0,532	28.3 (19.7)	29.6 (17.5)	0,783	26.4 (17.8)	29.3 (19.4)	0,507	21.5 (17.5)	29.7 (20.5)	0,108
	Incontinence aid				43.3 (27.4)	45.8 (33.3)	0,875	20.8 (24.8)	35.4 (32)	0,226	33.3 (17.8)	37.8 (34.3)	0,209	33.3 (47.1)	41 (32.8)	0,538
	Bowel symptoms				8.3 (17.3)	8.6 (13.7)	0,513	5.4 (6.8)	7.3 (11.3)	0,951	3.7 (7.1)	8.5 (13.3)	0,145	3.8 (6.5)	7.7 (12.8)	0,284
	Treatment symptoms				20.4 (16.6)	16.6 (12.9)	0,447	22.5 (13.3)	22.2 (18.3)	0,460	22.2 (16.9)	20 (16.9)	0,671	22 (16.9)	19 (17)	0,449
	Sexually active				26.2 (28.3)	31.9 (28.4)	0,420	25 (24.5)	35.3 (30.5)	0,179	16.7 (23.6)	37.3 (30.3)	0,006	28.2 (26.7)	37.5 (31.9)	0,398
	Sexual functioning				52.4 (28.3)	47.5 (25.7)	0,733	56 (20.8)	52.1 (20.5)	0,817	73.3 (26)	53.7 (19.4)	0,106	53.3 (15.1)	56.6 (20.4)	0,771
